# The Value of the Doctorate in Dentistry

**Published:** 1899-06

**Authors:** 


### Editorial.

### THE VALUE OF THE DOCTORATE IN DENTISTRY.

It has become a trite expression to assert that dentistry has grown to the proportion of a profession in the last half-century, and while this is true and the repetition of the fact becomes burdensome, it yet remains equally true that dentists, in this country, do not yet appreciate the fact that the degree of Doctor of Dental Surgery, established by the Baltimore College of Dental Surgery in 1839 (D.D.S.), and that of Harvard University D.M.D. (Dentariæ Medicinæ Doctor) represent an advance in dental culture that cannot be formulated in words.

These degrees have had the usual experience of all innovations in being contemned by those holding the older degrees and equally held in little respect by those in dentistry who for various causes had failed to acquire the honor.

For a long series of years the titles of D.D.S. and D.M.D. were not considered of sufficient importance to attract much attention outside of the schools conferring them, but as colleges increased and the graduates began to take prominent places in dental work, there arose two elements directly antagonistic, one to degrade the title and the other to make it more worthy of respect by environing it with legislative enactments. Both of these efforts have, positively and negatively, brought the two degrees into a critical contest in which there seems a possibility, remote it is true, of the titles being deprived of all value by being ground between the upper and lower millstones of law violation and law observance. It is, probably, true that the best test of the value of anything is the attempt to reproduce it. The counterfeiter and the plagiarist we will always have with us, and in spite of law, wherever money or credit is to be obtained, honestly or dishonestly, there will be minds capable of formulating methods for their accomplishment. It was, therefore, not at all surprising that as the degrees in dentistry became valuable the degenerate class saw their opportunity and took advantage of the demand by meeting it with diplomas that required only the engraver's skill and the printing-press to put them in circulation.

The first effect of legislative enactments naturally increased the value of these titles, and, as they represented more than formerly and required greater exertion to procure, they indirectly, but as positively, made the fraudulent trade more active and the degrees, to that extent, of less value.

This condition of affairs has not been apparent in this country; in fact, dentistry up to last year was quite content with its degrees, entirely unaware that a mine was being prepared by the Foreign Relations Committee of the Association of Faculties and which was sprung upon them at Omaha in 1898. It is true there were constant complaints from abroad of fraudulent diplomas, but these were supposed to have had their origin outside the limits of the United States. It was, therefore, a humiliating acknowledgment that this committee made, but the Faculties gave the energetic chairman full power, and the result of its work has been detailed upon our pages. One concern has been convicted and a stringent law has been passed in Illinois which, if enforced, will make it impossible for this work to be carried on in that State.

The effect of all this has had the result of stamping a stigma upon the degrees conferred in this country which it will take many years to overcome. This is more particularly noticeable in foreign countries.

This state of affairs has stirred the graduates of American colleges resident abroad into a state of activity never before witnessed. This is creditable and natural, for with a lessened respect for the diploma is a proportionate loss of prestige as American dentists. Never, in the experience of the writer, has more interest been taken abroad in this matter of degrees, and this is not confined to those of American birth, but is equally true of all those holding legitimate American degrees, and these are becoming very numerous throughout Europe. The reports that reach us of meetings held, papers read, and resolutions passed indicate that American dentists abroad mean to be heard upon this question. One or more delegates from foreign countries will meet with the Association of Faculties at its meeting at Niagara, and that body promises to have the most active meeting held since its organization.

The question of protection to the American degree, of whatever name, will demand intelligent and conservative treatment. Much that will be impossible of attainment will no doubt be presented, and much that ought to be adopted and enforced will equally be passed by, but it is hoped and believed that a wise judgment will prevail.

Suggestions of what ought to be done are flowing in from various sources. Our *confrères* abroad seem to be laboring under the impression that the remedy lies in a National diploma given by a National Board of Examiners and that such a document, or the endorsement of such a Board, would immediately change the opinion now held by the authorities of the various countries of Europe. It doubtless would change the present feeling of contempt to one of respect, but what then? Will the holder of that diploma, or endorsement, be any nearer recognition than he is to-day?

The editor of this journal is not near enough to European thought to give a positive opinion, but if experience is worth anything, it leans decidely to the view that, were a diploma endorsed by all the authority of the United States, it would not be recognized as entitling the holder to practise in competition with the title given in foreign countries, earned after periods of scholastic training under governmental laws and carried out by the ministers of instruction. It would seem, therefore, a waste of time to endeavor to accomplish the impossible. It is not so much the character of the diploma as it is the feeling of antagonism in foreign countries against all dentists holding the American degrees. Those of American birth are regarded as trespassers upon the rights of those to the manor born, and to the extent of their success are they, in their opinion, robbing them of a portion of their just dues. How this feeling can ever be overcome is a problem, and in the opinion of the writer it will ever remain a prominent factor in continuing the non-recognition now extant in Europe. This opposition is not unreasonable, and we in the United States have no cause to complain of this selfish side of human nature, for it is presented in our several States, depriving citizens of other States from entering into practice except by following prescribed laws, in many instances prohibitory in character.

Our friends abroad seem to be possessed with the idea that the government of the United States has the power to step in and meet the difficulty. They have, apparently, been so long within the influence of foreign thought that they have, in a measure, lost, to that extent, appreciation of American ideas of government. The Constitution of the United States distinctly states what the government can and cannot do. The rights of the several States have always been jealously guarded, and the duties of each of these to provide laws for their internal government cannot be infringed upon. This limitation of power is one of the peculiarities of our system, difficult of comprehension by foreign powers, and instances are numerous where indemnity for injuries has been demanded and not complied with, for the reason that they have been outside of the national jurisdiction. The power to create a National Board of Examiners does not exist, and to effect it a radical change must be made in the Constitution, and this, it is safe to assume, will not be done.

If this be correct, the true idea is to conserve the methods at our command and endeavor to surround the diploma, as now given, with all the safeguards of State laws, intelligently framed and with equal intelligence enforced.

The question has assumed such proportions that the limits of a single editorial are insufficient to meet it at all points. Its importance is, however, fully recognized, and its free discussion will help to clear up much confusion that seems to exist not only abroad, but in this country.

The degree, honorably earned, whether held in this country or abroad, has a value recognized by all, and whatever shadow may have been thrown upon it by disreputable practices will be dispelled by the sure educational foundation which is being continually strengthened through a broader culture. Upon this must depend the value of any degree, and in proportion as this is increased will the doctorate in dentistry be respected.

